# Modelling the effectiveness of intervention strategies to control COVID-19 outbreaks and estimating healthcare demand in Germany

**DOI:** 10.1016/j.puhip.2021.100121

**Published:** 2021-04-19

**Authors:** Sudarat Chadsuthi, Charin Modchang

**Affiliations:** a Department of Physics, Research Center for Academic Excellence in Applied Physics, Faculty of Science, Naresuan University, Phitsanulok, 65000, Thailand; b Biophysics Group, Department of Physics, Faculty of Science, Mahidol University, Bangkok, 10400, Thailand; c Centre of Excellence in Mathematics, CHE, 328, Si Ayutthaya Road, Bangkok, 10400, Thailand

**Keywords:** COVID-19, Hospitalization, Active case-finding, Self-isolation, Physical distancing

## Abstract

**Objectives:**

An outbreak of the novel coronavirus in December 2019 caused a worldwide pandemic. This disease also impacts European countries, including Germany. Without effective medicines or vaccines, non-pharmaceutical interventions are the best strategy to reduce the number of cases.

**Study design:**

A deterministic model was simulated to evaluate the number of infectious and healthcare demand.

**Method:**

Using an age-structured SEIR model for the COVID-19 transmission, we project the COVID-19–associated demand for hospital and ICU beds within Germany. We estimated the effectiveness of different control measures, including active case-finding and quarantining of asymptomatic persons, self-isolation of people who had contact with an infectious person, and physical distancing, as well as a combination of these control measures.

**Results:**

We found that contact tracing could reduce the peak of ICU beds as well as mass testing. The time delay between diagnosis and self-isolation influences the control measures. Physical distancing to limit the contact rate would delay the peak of the outbreak, which results in the demand for ICU beds being below the capacity during the early outbreak.

**Conclusions:**

Our study analyzed several scenarios in order to provide policymakers that face the pandemic of COVID-19 with insights into the different measures available. We highlight that the individuals who have had contact with a virus-positive person must be quarantined as soon as possible to reduce contact with possible infectious cases and to reduce transmission. Keeping physical distance and having fewer contacts should be implemented to prevent overwhelming ICU demand.

## Highlights

• We developed an age-structured SEIR model for the COVID-19 to project the demand for hospital and ICU beds within Germany.
• The self-isolation of at-risk groups and mass testing have impacts on the peak of ICU beds.
• Physical distancing would delay the peak of outbreak.

## 1 Introduction

In December 2019, the novel coronavirus (severe acute respiratory syndrome coronavirus 2 (SARS-CoV-2)) was first identified in Wuhan, China, and then rapidly spread across the country [1,2]. The name of the new coronavirus disease was announced as COVID-19 on 11 February 2020 by the World Health Organization (WHO). Without natural immunity, this disease has spread worldwide to more than 200 countries and over 3 million cases. In the European countries, on 24 January 2020, the first three cases were found and identified in France [3,4]. In Germany, four cases with indirect links to Wuhan were reported on 28 January [3]. After that, COVID-19 cases have increased up to 160,000 cases by 5 May.

To control the outbreak, non-pharmaceutical interventions involving isolation, contact tracing, and social distancing were introduced. The effectiveness of case isolation depends on the time delay from symptom onset to isolation [5]. Contact tracing of those who have symptoms and prompt isolation could reduce and control the outbreak [5]. During the early phase of the COVID-19 outbreak, the basic reproduction number was around 2–3 [6,7], and the proportion of asymptomatic cases was high more detail [[8], [9], [10]]. Using only contact tracing and symptomatic case isolation may not be an effective strategy as asymptomatic cases will still spread the virus leading to an outbreak again. Some countries have used mass testing combined with isolation of infected cases and tracing and quarantining those who have had contact with infectious people, such as South Korea and Germany [11]. Learning from severe acute respiratory syndrome (SARS) in 2003, Hong Kong and Singapore responded by aggressive testing, isolating infected people, and tracing and quarantine infected contacts [12]. Physical distancing or school closures have also been studied to reduce the number of infections [13,14]. However, it is unclear that implementation of strict physical distancing such lockdown alone can control the outbreak of COVID-19. To effectively reduce the infections, the intensity, duration, and timing of different mitigation scenarios on the transmission dynamics need to be investigated.

Mathematical modeling can be used to estimate the effectiveness of control measures for many infectious diseases. The modified Susceptible-Exposed-Infectious-Recovery (SEIR) model was used to predict the epidemics peaks and sizes for COVID-19 in China [15]. A well-mixed SEIR compartmental model was also applied to study the effectiveness of quarantine in Wuhan city [16]. Wu et al. also used a modified SEIR model that considered air transportation information to estimate the number of exported cases from Wuhan to other cities outside China [17]. Prem et al. studied the effect of control strategies to reduce social mixing using the SEIR model [13]. Although there were many models that studied the impact of non-pharmaceutical interventions, none of them focused on investigating the age-specific demand for hospital and ICU beds [[18], [19], [20]]. In this work, we developed an age-structured SEIR model for investigating the age-specific demand for hospital and ICU beds in Germany. In addition, we also investigated several combinations of intervention strategies, e.g., active case-finding, self-isolation, physical distancing, and cyclic lockdown, to reduce the impact of the COVID-19 outbreak. Our modelling results may provide a guideline to control and keep the healthcare services demand below the capacity.

## 2 Methods

### 2.1 SEIR model

We modified the SEIR model to study the dynamics of COVID-19 transmission (Fig. 1). The population is divided into 10 compartments, each with 16 age groups (5-year bands) [21]. We classify the status of the population into five infection states, susceptible (S), exposed but not yet infectious (E), infectious (I), and recovery (R) or die (D). To implement self-isolation and active case-finding strategies, the exposed state will become self-quarantine, who had contact with an infectious individual and quarantine themselves before infectiousness ($Q^{s}$) with a probability of self-quarantine (y), clinical infectious, those who have severe symptoms and need treatment ($I^{c}$) with a probability of clinical symptoms for age group i ($m_{i}$) and, sub-clinical infectious, who are infectious but do not have any symptoms or have only mild symptoms and do not require treatment ($I^{sc}$). We assumed that the infectious individuals with severe symptoms ($I^{c}$) will need hospitalization (H) or critical care in an ICU ($I^{ICU}$) with a probability of a clinical case in ICU bed ($h_{i}$). In this model, ICU cases can either die (*D*) with a death rate of ICU patients ($d_{c}$) or be discharged and remained hospitalized (*H*) until recovery [22]. We assumed that the infectious individuals with severe symptoms will quarantine themselves and go to a hospital, and self-quarantine individuals will quarantine themselves as soon as with delay from exposure to self-isolation ($1/\tau_{s}$). Thus, active case-finding with testing will only look for asymptomatic individuals with positive results that will be isolated in their homes ($Q^{sc}$). We assumed that Germany is a closed system and well-mixed with a constant population size of 83.5 million [21]. The system of equations for the age group i can be described by

$$\frac{dS_{i}}{dt}=-\beta S_{i}\overset{n}{\underset{j=1}{\sum }}c_{ij}\left(I_{j}^{c}+fI_{j}^{sc}\right)/N_{j}$$

$$\frac{dE_{i}}{dt}=\beta S_{i}\sum_{j=1}^{n}c_{ij}\left(I_{j}^{c}+fI_{j}^{sc}\right)/N_{j}\;-\left(1-y_{i}\right)m_{i}\sigma E_{i}-\left(1-y_{i}\right)\left(1-m_{i}\right)\sigma E_{i}-y_{i}\tau_{s}E_{i}$$

$$\frac{dI_{i}^{c}}{dt}=\left(1-y_{i}\right)m_{i}\sigma E_{i}-h_{i}\omega I_{i}^{c}-\left(1-h_{i}\right)\omega I_{i}^{c}$$

$$\frac{dI_{i}^{sc}}{dt}=\left(1-y_{i}\right)\left(1-m_{i}\right)\sigma E_{i}-\delta_{i}\tau_{a}I_{i}^{sc}-\left(1-\delta_{i}\right)\gamma I_{i}^{sc}$$

$$\frac{dQ_{i}^{s}}{dt}=y_{i}\tau_{s}E_{i}-m_{i}h_{i}\rho Q_{i}^{s}-m_{i}\left(1-h_{i}\right)\rho Q_{i}^{s}-\left(1-m_{i}\right)\gamma_{s}Q_{i}^{s}$$

$$\frac{dI_{i}^{ICU}}{dt}=h_{i}\omega I_{i}^{c}+m_{i}h_{i}\rho Q_{i}^{s}-\varphi_{c}\left(1-d_{c}\right)I_{i}^{ICU}-\mu_{c}d_{c}I_{i}^{ICU}$$

$$\frac{dH_{i}}{dt}=\left(1-h_{i}\right)\omega I_{i}^{c}+m_{i}\left(1-h_{i}\right)\rho Q_{i}^{s}+\varphi_{c}\left(1-d_{c}\right)I_{i}^{ICU}-\varphi_{h}H_{j}$$

$$\frac{dQ_{i}^{sc}}{dt}=\delta_{i}\tau_{a}I_{i}^{sc}-\gamma_{sc}Q_{i}^{sc}$$

$$\frac{dR_{i}}{dt}=\left(1-\delta_{i}\right)\gamma I_{i}^{sc}+\left(1-m_{i}\right)\gamma_{s}Q_{i}^{s}+\varphi_{h}H_{j}+\gamma_{sc}Q_{i}^{sc}$$

$$\frac{dD_{i}}{dt}=\mu_{c}d_{c}I_{i}^{ICU}$$

**Fig. 1:**
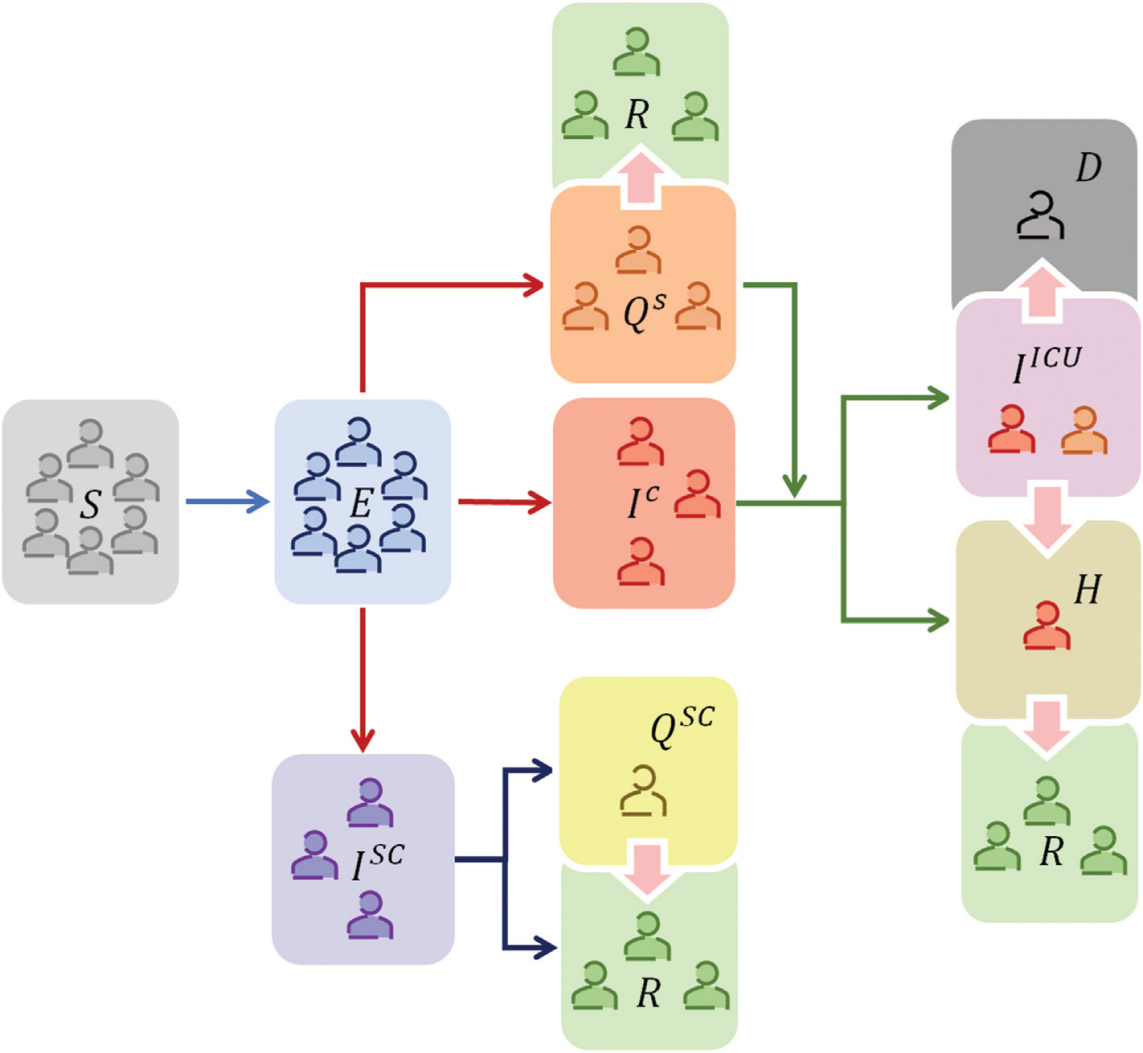
Flow diagram of the model. The infectious state of an age group is subdivided into three classes, namely, clinically infectious ($I^{c}$), sub-clinically infectious ($I^{sc}$), and infectious with self-isolation ($Q^{s}$). Only infectious individuals with severe symptoms ($I^{c}$) will need hospitalization (H) and critical care in ICU ($I^{ICU}$).

### 2.2 Model calibration

We used parameters from the literature described in Table 1. The initial cases were estimated to fit with the outbreak in Germany [34]. The transmission rate (β) was calculated based on the time-varying reproduction numbers ($R_{t}$) of Germany from 2 March to 23 March 2020. The estimated $R_{t}$ was calculated using the EpiEstim R package [35] with a mean serial interval of 6.6 days and a standard deviation of 4.88 days [36].

**Table 1:** Description of parameters.

parameter	description	value	References
β	Transmission rate	calculated from $R_{t}$	–
$c_{\mathrm{ij}}$	Contacts of age group j made by age group i	estimated	[23]
$m_{i}$	Probability of clinical symptoms for age group i	estimated	[8]
$h_{i}$	Probability of a clinical case in ICU bed for age group i	estimated	[24]
1/σ	Latent period (days)	2.9	[[25], [26]] [[,^26^]
$1/\tau_{s}$	Delay from exposure to self-isolation (days)	1-2 (varied)	–
1/ρ	Delay from symptoms onset to hospitalization from self-isolation (days)	$1/\sigma +1/\omega -1/\tau_{s}$	–
1/ω	Delay from symptom onset to hospitalization (days)	4.64	[27,28]
$1/\tau_{a}$	Delay of testing results (days)	1-2 (varied)	–
$1/\varphi_{c}$	Duration of ICU stay (days)	9	[29]
$1/\varphi_{h}$	Duration of hospitalization (days)	10	[30]
1/γ	Duration of infectiousness (days)	7	[25,31]
$1/\gamma_{\mathrm{sc}}$	Duration of subclinical who isolation before recovery (days)	$1/\gamma -1/\tau_{a}$	–
$1/\gamma_{s}$	Duration of subclinical who self-isolation before recovery (days)	$1/\sigma +1/\gamma -1/\tau_{s}$	–
$1/\mu_{c}$	Duration from ICU admission death (days)	7	[29,32]
$d_{c}$	Death rate of ICU patients	0.007	[33]
δ(t)	Fraction of testing	varied	–
y(t)	Probability of self-quarantine	varied	–
f	Relative infectiousness of subclinical cases	0.5	[26]

### 2.3 Intervention analysis

For the baseline (no intervention), we set the time-varying reproduction numbers ($R_{t}$) of Germany from 2 March to 23 March 2020. After that, the reproduction number was randomly selected from a Normal distribution with a mean of 2.08 and a standard deviation of 0.01 and then held constant after 24 March 2020. We simulated 200 independent models over 2 years. The intervention scenarios, i.e., active case-finding, self-isolation, physical distancing, and their combination, were started on 24 March 2020 as we estimated a two-day delay from when the restrictions were issued on 22 March.

Active case-finding was defined as the mass testing campaign of asymptomatic or mild symptom cases ($I^{sc}$) and then limiting contact with these infectious individuals by isolating and avoiding contact with the community. We assumed that individuals during the latent period would not test positive [37]. The time delay from testing to getting the results ($1/\tau_{a}$) was studied with a one- or two-day delay. Using contact tracing, individuals who have contact with an infectious person can be quarantined by themselves with no other contact. Public health officials can promote this self-isolation scenario to help reduce the impact of the COVID-19 outbreak. We measured the effectiveness of contact tracing using a time delay from the infectiousness in a person to informing their contacts to be within one or two days ($1/\tau_{s}$). Physical distancing or social distancing is one of the suggestion scenarios. In this work, we considered different scenarios for reducing contact, such as the duration of the lockdown (less contact), reducing contacts by half, and a cyclic scheduled lockdown [38]. A combination of interventions was considered and proposed to prevent overloading of the medical system.

## 3 Results

We first estimated the reproduction number (Fig. 2) from 2 March to 23 March to fit the rate of increase in infected cases. During the early outbreak, the reproduction number ($R_{t}$) was about 4–6, which indicates a large outbreak. After that, the reproduction number decreases as awareness of the pandemic increases. On day 22 (24 March), $R_{t}$ is reduced when the lockdown strategy was issued. We calibrated the parameters based on $R_{t}$ to fit with the observed data (Fig. 2). Without any interventions, the simulation as a baseline provided the cumulative incidence of all infection compartments of 22 million with the attack rate of about 26%. We estimated the number of hospitalization and ICU admission (Table 2). We found that the infectious individuals aged 25 to 54, referring to people in their prime working lives, will need 78 ICU beds per 1000 population (median of 200 simulations) more than infected individuals over 55 years 2.59 times due to the working people have high contact.

**Fig. 2:**
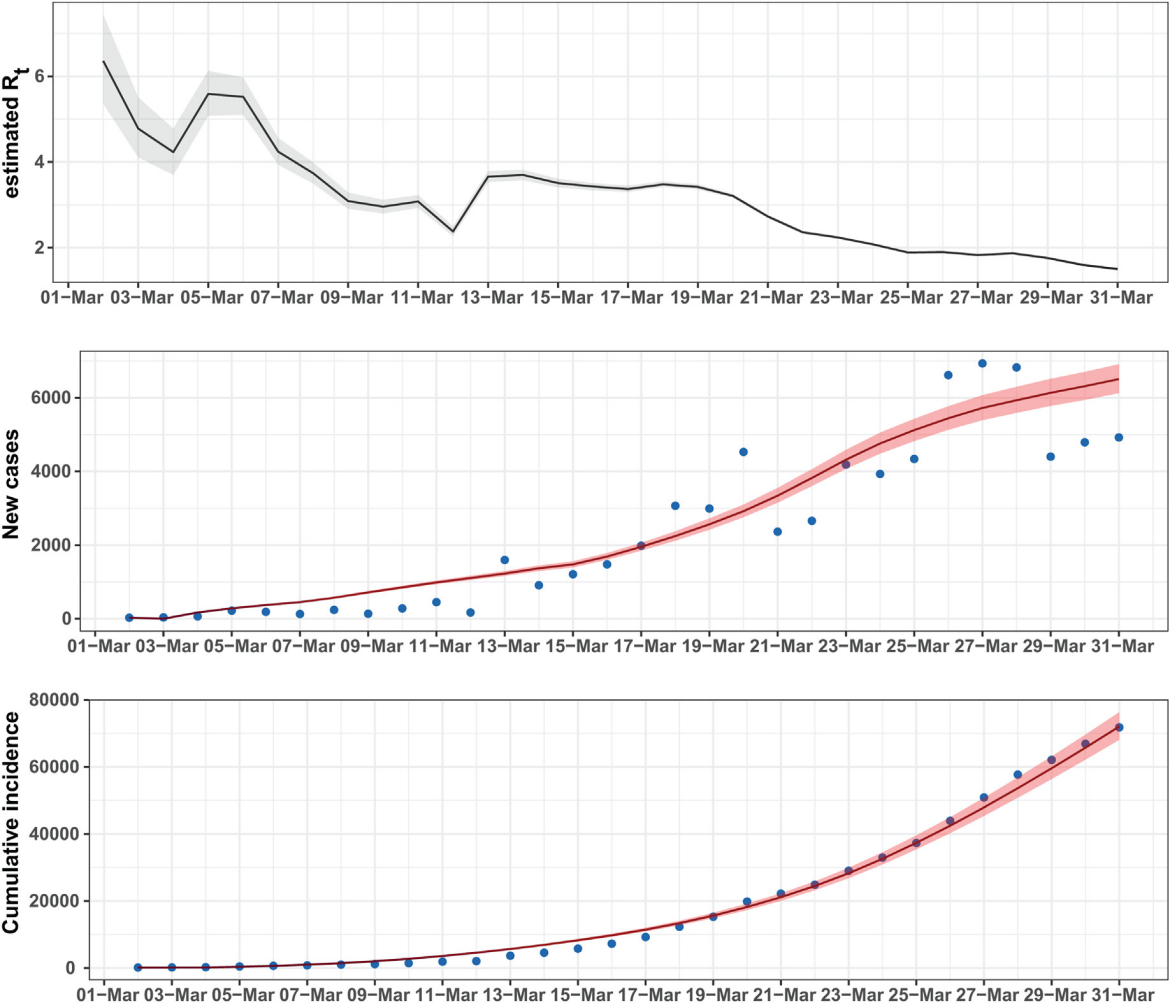
Plots of the estimated reproduction number (a), number of daily new cases (b), and cumulative incidences (c). The dots show the observed data, and the solid lines represent the simulation results. The shaded area indicates the 95% confidence interval.

**Table 2:** Testing scenarios. The estimated number of total incidences, peak capacity requirement of non-ICU and ICU beds, and time to peak.

Scenarios	Total incidences	Requirement at peak		Time to peak in days	
		Non-ICU	ICU	Non-ICU	ICU
Base	22.0 ​M	832 k	118 k	192	190
$1/\tau_{a}=2$					
0.01	21.3 ​M	786 k	111 k	194	192
0.02	20.6 ​M	742 k	105 k	196	195
0.05	18.8 ​M	626 k	87.5 k	202	201
0.10	16.1 ​M	473 k	65.8 k	212	211
0.20	11.9 ​M	273 k	37.6 k	229	227
0.50	4.72 ​M	71.2 k	9.85 k	176	175
$1/\tau_{a}=1$					
0.01	20.3 ​M	726 k	102 k	197	195
0.02	18.9 ​M	634 k	89.2 k	202	200
0.05	15.1 ​M	423 k	59.3 k	216	215
0.10	10.5 ​M	220 k	30.7 k	233	231
0.20	5.01 ​M	75.6 k	10.5 k	188	186
0.50	1.39 ​M	47.0 k	6.63 k	49	44

**Fig. 3:**
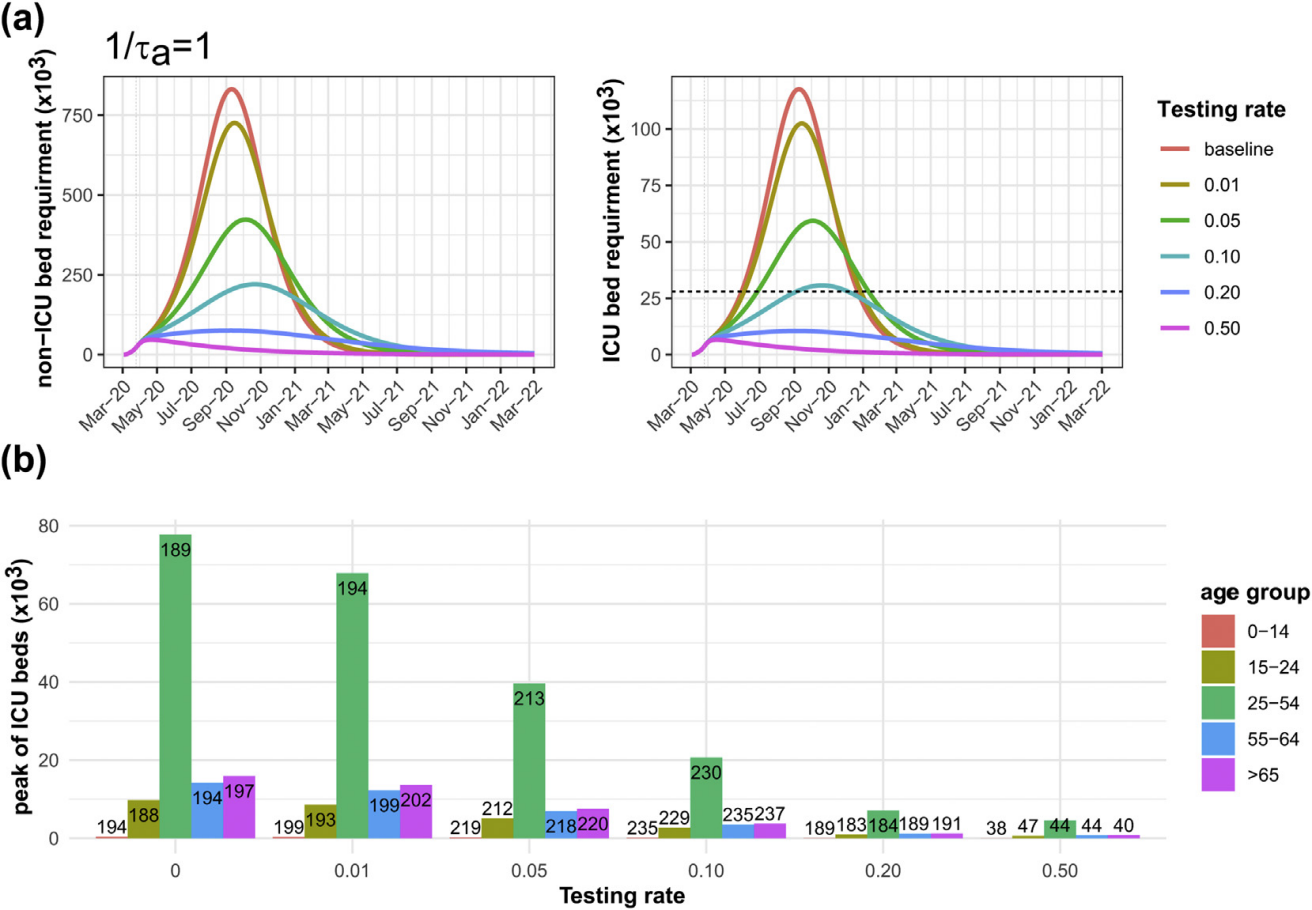
A 1-day delay of testing results. Simulation outcomes for difference testing rate of non-ICU and ICU bed requirements compared to baseline (a) and age-specific ICU bed requirements at peak with time to peak indicated in the bars (b). The black dashed lines represent the total of 28,000 ICU beds.

Assuming that public health agencies have sufficient testing capacity for COVID-19, we varied mass random-testing from 1% to 50% of the population (Table 2). For a 1-day delay in getting the testing results, we found the total number of incidences was reduced from baseline for an increasing testing rate. The effectiveness 50% testing rate of a 2-day delay to get the testing results could not reduce the number of cases as well as a 1-day delay, as it caused a 79% reduction of baseline compared to a 1-day delay, it caused 94% reduction of baseline. At every testing rate, the curve of incidences, non-ICU bed usage, and ICU bed usage was flatter with a 1-day delay in testing results (Fig. 3) compared to a 2-day delay (Supplementary Figure 1). To present the results of age-specific ICU admission, we grouped the 16 age groups into 5 age groups according to employment rates. People aged less than 15 represent the non-worker group, people aged 15 to 24 represent the labour group following education, people aged 25 to 54 represent a prime working group, people aged 55 to 64 represent the passing of peak working group, and people aged more than 64 represent retired [39]. For a 1-day delay in the testing results, the results of the age-specific ICU admission (Fig. 3) showed a peak of infectious patients of worker group (aged 25–54) higher than the other groups and required ICU beds exceeded the total capacity when the testing less than 20%. Our results suggested that the efficiency of testing has an impact on control measures. Using the best testing parameters, such as 50% of the population and a 1-day delay, the number of ICU beds reduced to about 6630 beds. However, a high testing rate costs a lot of resources.Where M is a million unit ( $\times 10^{6}$) and k is a thousand unit ($\times 10^{3}$)

**Fig. 4:**
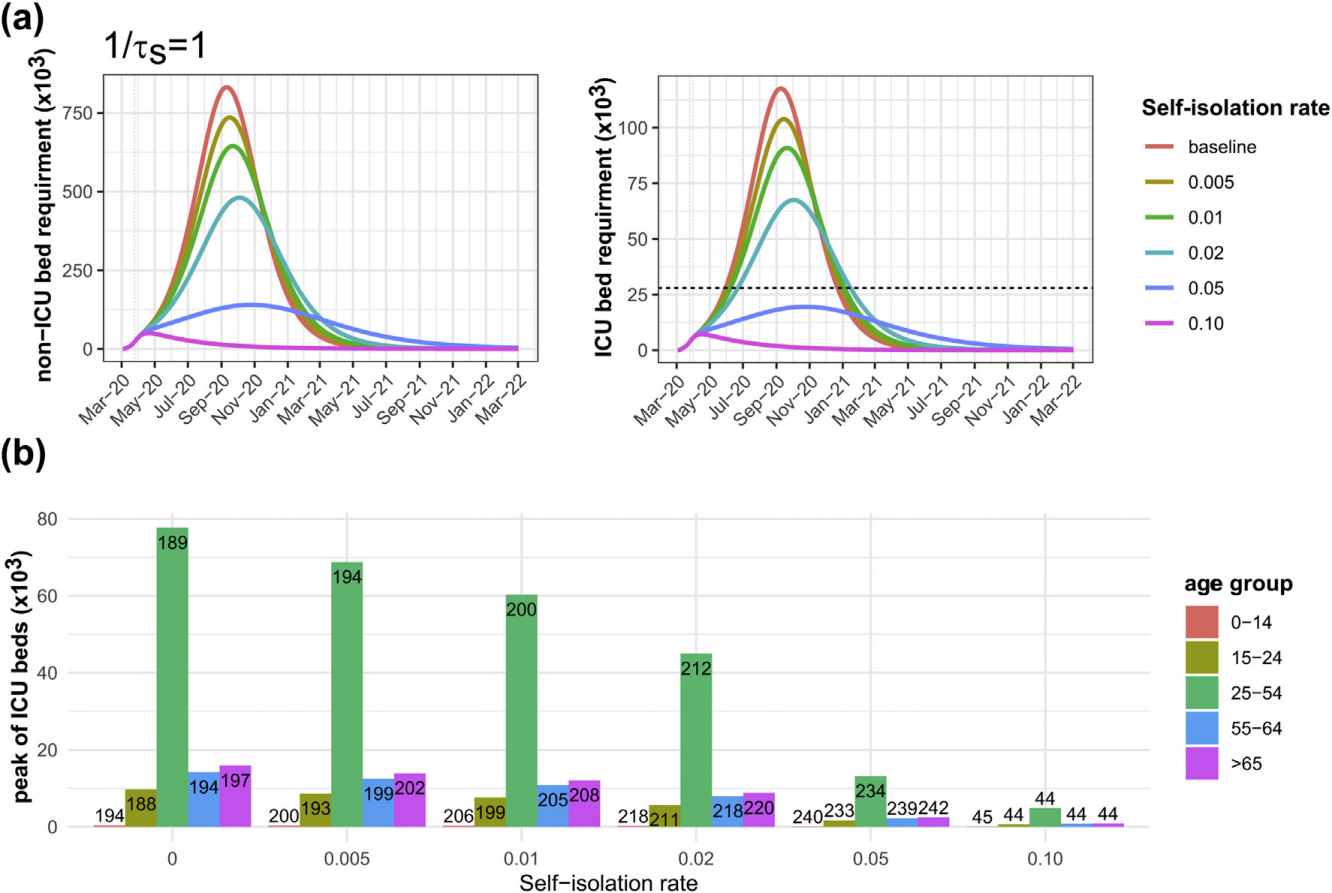
A 1-day delay of self-isolation. Simulation outcomes for difference self-isolation rates on non-ICU and ICU bed requirements compared to baseline (a) and age-specific ICU bed requirements at peak with time to peak indicated in the bars (b). The black dashed lines represent the total of 28,000 ICU beds.

If contact tracing of infected individuals was implemented, individuals who have been in contact with infectious people may isolate themselves and make no more contact with others. We varied the plausible rate of self-isolation from 0.005 to 0.10 (Table 3). We found that a 1% self-isolation rate 1-day after exposure to an infectious person could reduce the total incidences by 14%. The peak of ICU bed usage dropped below the total capacity [40] when more than 5% of possible exposed individuals’ self-isolation 1 day after contact with an infectious person (Fig. 4). The age of the ICU admission patients is also shown in Fig. 4. We observed similar trends for lower self-isolation rates compared to a higher testing rate. The results of self-isolation 2 days after contact with an infectious person also showed in Supplementary Figure 2. The results indicate testing only a small fraction of the population or self-isolation could not keep the ICU demand below the threshold and could not delay the outbreak for healthcare service preparation.Where M is a million unit ( $\times 10^{6}$) and k is a thousand unit ($\times 10^{3}$)

**Table 3:** Self-isolation scenarios. The estimated number of total incidences, peak capacity requirement of non-ICU and ICU beds, and time to peak.

Scenarios	Total Incidence	Requirement at peak		Time to peak in days	
		Non-ICU	ICU	Non-ICU	ICU
Base	22.0 ​M	832 k	118 k	192	190
$1/\tau_{s}=2$					
0.001	21.8 ​M	822 k	116 k	192	191
0.005	21.2 ​M	783 k	111 k	195	193
0.01	20.5 ​M	734 k	104 k	198	196
0.02	19.1 ​M	641 k	90.4 k	204	202
0.05	14.7 ​M	395 k	55.4 k	224	223
0.10	7.24 ​M	120 k	16.8 k	236	234
$1/\tau_{s}=1$					
0.001	21.6 ​M	812 k	115 k	193	191
0.005	20.4 ​M	736 k	104 k	197	196
0.01	18.9 ​M	645 k	90.9 k	203	201
0.02	16.0 ​M	480 k	67.5 k	215	214
0.05	7.58 ​M	140 k	19.5 k	237	235
0.10	935 k	50.0 k	7.12 k	48	44

**Fig. 5:**
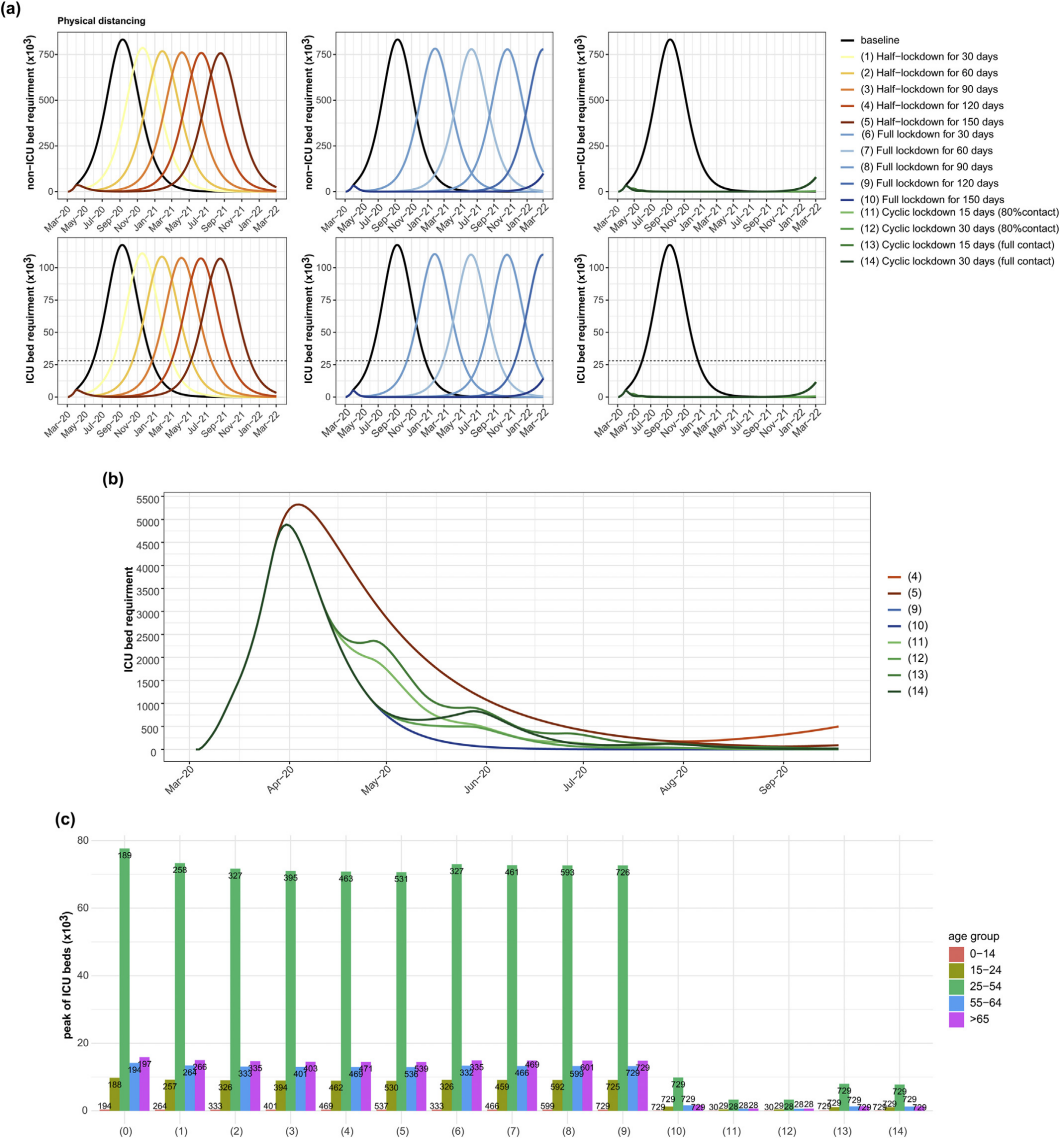
The epidemic curve under different physical distancing scenarios. The plot of non-ICU and ICU bed requirement compared to baseline (a) and during the early outbreak (b) and the age-specific ICU bed requirements at peak with time to peak indicated in the bars (c).

Physical distancing is a control measure that can reduce contact between people, reducing the transmission and delaying the peak of the outbreak. In this work, we simulated several scenarios (Table 4), where the epidemic curve and age-specific ICU bed usage are shown in Fig. 5. Our results suggest that only reducing contacts by half for 30 days could delay the peak of ICU beds for 70 days. However, this control measure could reduce the total incidences by only 1.4% of the baseline. Comparing a half lockdown with a full lockdown, we found that the total number of incidences and hospitalization is not different, but a full lockdown could more delay the peak of ICU beds. We also found that cyclic lockdown could reduce the incidence rate more than a single full lockdown. Cyclic lockdowns with an equivalence number of locked-down working days, with either half-lockdowns or full lockdowns for long periods, could delay the peak of infected cases. Our results also suggest that reducing 20% of contacts during the relaxation of a cyclic lockdown could reduce the incidences and delay the peak more than full relaxation. We also considered the curve of ICU bed usage during the early outbreak of some scenarios (Fig. 5 (b)). Considering the short-term effects, a full lockdown of 150 days is the best strategy to control the demand for health care services. However, a full lockdown for 3 months would bring additional problems such as economic and social distress, compared to a cyclic lockdown.Where M is a million unit ( $\times 10^{6}$) and k is a thousand unit ($\times 10^{3}$)

**Table 4:** Physical distancing scenarios. The estimated number of total incidences, peak capacity requirement of non-ICU and ICU beds, and time to peak.

Scenarios	Total Incidence	Requirement at peak		Time to peak in days	
		Non-ICU	ICU	Non-ICU	ICU
Base	22.0 ​M	832 k	118 k	192	190
Half-lockdown for 30 days	21.7 ​M	786 k	111 k	261	260
24-Mar to 22 Apr (50% of work, school and other contacts)					
Half-lockdown for 60 days	21.6 ​M	768 k	109 k	330	329
24-Mar to 22 May (50% of work, school and other contacts)					
Half-lockdown for 90 days	21.5 ​M	761 k	108 k	398.5	397
24-Mar to 21 June (50% of work, school and other contacts)					
Half-lockdown for 120 days	21.5 ​M	758 k	107 k	466	465
24-Mar to 21 July (50% of work, school and other contacts)					
Half-lockdown for 150 days	21.4 ​M	757 k	107 k	534	532
24-Mar to 20 August (50% of work, school and other contacts)					
Full lockdown for 30 days	21.6 ​M	782 k	111 k	330	328
24-Mar to 22 Apr (10% of work, school and other contacts)					
Full lockdown for 60 days	21.6 ​M	779 k	110 k	464	462
24-Mar to 22 May (10% of work, school and other contacts)					
Full lockdown for 90 days	21.1 ​M	778 k	110 k	596	595
24-Mar to 21 June (10% of work, school and other contacts)					
Full lockdown for 120 days	12.8 ​M	778 k	110 k	729	727
24-Mar to 21 July (10% of work, school and other contacts)					
Full lockdown for 150 days	1.14 ​M	99.2 k	14.4 k	729	729
24-Mar to 20 Aug (10% of work, school and other contacts)					
Cyclic lockdown 15 days until 17 Jan 21 (repeated lockdown and 80% of work, school and other contacts for 15 days)	263 k	32.0 k	4.89 k	29	28
Cyclic lockdown 30 days until 17 Jan 21 (repeated lockdown and 80% of work, school and other contacts for 30 days)	235 k	32.0 k	4.89 k	29	28
Cyclic lockdown 15 days until 17 Jan 21 (repeated lockdown and full contact for 15 days)	1.04 ​M	80.5 k	11.7 k	729	729
Cyclic lockdown 30 days until 17 Jan 21 (repeated lockdown and full contact for 30 days)	974 k	78.2 k	11.4 k	729	729

**Fig. 6:**
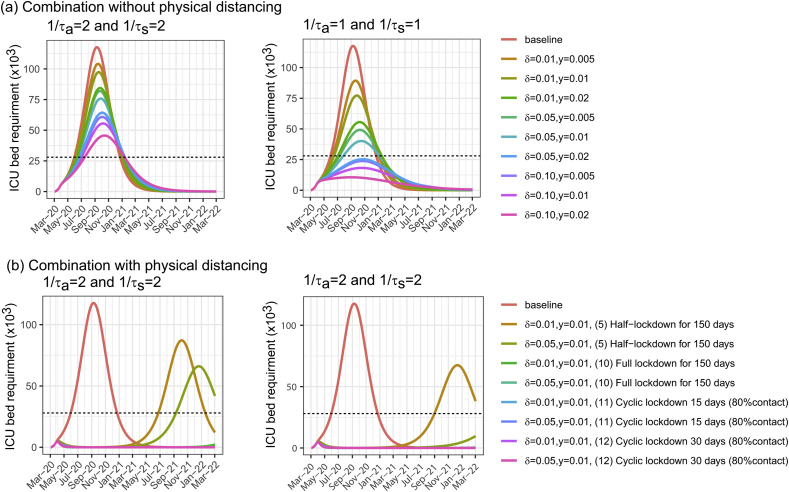
The epidemic curve and ICU bed requirement with different combination scenarios. The upper figure (a) is the results of a combination of testing rate and self-isolation rate, and the lower figure is the results of a combination of three scenarios.

Thus, our results highlighted testing and self-isolation scenarios with a high rate could reduce the healthcare demand and the total incidence. The physical distancing could only help in delay the peak of the outbreak. Without physical distancing, we estimated the effectiveness of combined testing and self-isolation at reasonable rates (Fig. 6 (a) and Supplementary Table 1). We found that 5% of testing rate and 2% of self-isolation could reduce the healthcare demand under the hospital capacity for a 1-day delay.

Scenarios with different combinations of testing, self-isolation, and physical distancing were considered to estimate the requirement for ICU beds (Fig. 6 (b) and Supplementary Table 2). For a 1-day delay with 5% of the mass random testing rate, a contact tracing system (with 1% self-isolation) and a half-lockdown for 150 days could reduce the number of ICU beds below the threshold for 2 years simulation. The scenarios with cyclic lockdowns could delay a large outbreak for longer periods. Overall, our results showed the most effective strategy is the full lockdown or cyclic lockdown to delay the peak of cases, including testing and self-isolation.

## 4 Discussion

Due to the worldwide spread of COVID-19 and the increasing number of cases, including in Germany, strategies to control and reduce the impact of this outbreak are necessary, as well as models to estimate the healthcare demand. In this work, we modified an age-structured SEIR model and used it to study the effectiveness of active case-finding, self-isolation and physical distancing, and combinations of multiple strategies. Without any interventions, high demand for health care services will occur and overload the system. Having sufficient ICU beds for severe cases is an important factor to save the lives of the patients who have severe respiratory failure and need mechanical ventilation for treatment [22,41]. In Germany, before the outbreak of COVID-19, there were about 28,000 intensive care beds, which are around 34 per 100,000 people [40]. However, about 80% of ICU beds are routinely occupied [42], which means that there are about 5,600 beds available.

There is some evidence suggesting that asymptomatic cases and sub-clinical cases with mild symptoms are potential sources of COVID-19 infection [[43], [44], [45]]. We estimated the effectiveness of mass testing for sub-clinical or asymptomatic cases as an active case-finding campaign. Our results suggest using mass testing could reduce the incidence peak, which agrees with findings from the work of Giordano and co-workers[46]. Using fast diagnostic tests could reduce a greater number of cases than slower tests. This level of a testing campaign would use a lot of tests, which also means spending a lot and cannot test all of the population. However, we can limit the testing for only the individuals who are at risk with the help of tracing, which could use a smaller number of tests. Using a rapid test with high sensitivity and specification is required to reduce the false negative rates. For example, a rapid test with 99% sensitivity and 95% specificity would give a false negative result, which is defined as a person having the disease but receiving a negative result, of 1%. Using nucleic acid tests such as polymerase chain reaction (PCR) could provide more accurate results, but this method is more expensive for sample acquisition, preparation, and device operation [47]. However, this strategy can be targeted to high risk groups, such as healthcare workers who are more likely to be exposed to infectious persons [44] and could help end the epidemic [48]. Mass testing could also provide important data to understand the infection dynamics, such as infection rate and duration of symptoms, which could improve the epidemiological models.

We also examined different scenarios of self-isolation of people in contact with an infectious person. Public health officials can promote a self-isolation campaign using a tracing application, such as a mobile application, or manual tracing in order to provide the timing and location where infected cases were found. Self-isolation with contact tracing could help prevent the transmission of the virus by isolating possibly infectious persons before they can spread the disease [5]. We found that a self-isolation rate of only 5% could bring down the peak of ICU bed usage more than mass testing of 10% of the population, as supported by the work of Kucharski and co-worker [49]. However, this strategy is less effective when there is a high rate of transmission before symptom onset [5]. This strategy also requires a suitable system to quickly trace individuals and to identify the precise location of infectious cases. Using both mass testing and self-isolation together could reduce the number of infected cases more than either used alone. These strategies successful in control the early COVID-19 outbreak in Korea [50].

The time delay between diagnosis as infectious and self-isolation influences the effectiveness of these control measures. For example, a 50% testing rate with a 1-day delay could reduce the peak of ICU admission compared to a 2-day delay. 10% of people self-isolating within 24 ​h after exposure to an infectious individual could reduce the ICU bed usage peak by twice compared to a 48-h delay in isolating. Thus, timely testing results and self-isolation should be considered a key method to control the demand for ICU beds.

To delay the peak of the epidemic, we analyzed some scenarios for physical distancing. Our results suggest that a half lockdown or full lockdown for a short period of time results in a delay of incidences and the ICU peak demand, which is consistent with previous findings [13,46]. However, it cannot reduce the peak of incidences as well as active case-finding and self-isolation strategies, when the lockdown was lifted as the number of incidences quickly raised again. Using a lockdown to suppress COVID-19 will have economic and social costs such as lost jobs. Another strategy for the lockdown was a cyclic lockdown proposed by Karin and co-worker [38]. We investigated some cyclic lockdown strategies and compared them with a full lockdown with an equal number of locked down days. Our model results in a delay of the ICU peak for the cyclic lockdowns till much later. Thus, we could use the cyclic lockdown as an alternative strategy for longer periods, with less economic problems, as it replaces full employment with part-time jobs instead of unemployment [38]. An additional campaign such as wearing a mask or washing hands could raise the awareness of contact between people. Most of the models added a mask variable by reducing the transmission rate from infected individuals [[51], [52], [53]].

This work has some limitations; our mathematical model did not consider school closures as students may make contact even while schools are closed. As we could not separate the number of imported infected individuals from the number of locally infected individuals, the estimated time-varying reproduction number might not well represent the transmission dynamics in Germany. Our results are based on the parameters of the initial outbreak from the literature of serval countries, which could cause some estimation errors. However, we calibrated the initial parameters with the observed data in Germany. Our models can also be re-simulation once updated data is available to more accurately estimate the response.

Our study analyzed several scenarios to control the pandemic of COVID-19 and to inform on how to best affect the situation. Healthcare services must be prepared with sufficient non-ICU and ICU beds to meet the demand. Governments, hospital administrators, and public health agencies must carefully use some mitigation measures in order to keep businesses running and the demand for ICU cases below the ICU bed capacity. Our results highlight that the individuals who have had contact with infectious cases must be quarantined as soon as possible to reduce the transmission rate. Keeping physical distance with fewer contacts during a lockdown should be issued to not produce overwhelming ICU demand in the absence of herd immunity or until vaccination is available. Our results can help in understanding the dynamics of disease transmission and intervention strategies. This modified SEIR model may apply to other diseases which have similar transmission, such as influenza. However, using any non-pharmaceutical interventions takes some costs and economic losses. Using vaccination with suitable strategies should consider in the future.

## Declaration of competing interest

The authors declare no conflicts of interest.

## Figures and Tables

**figs1:**
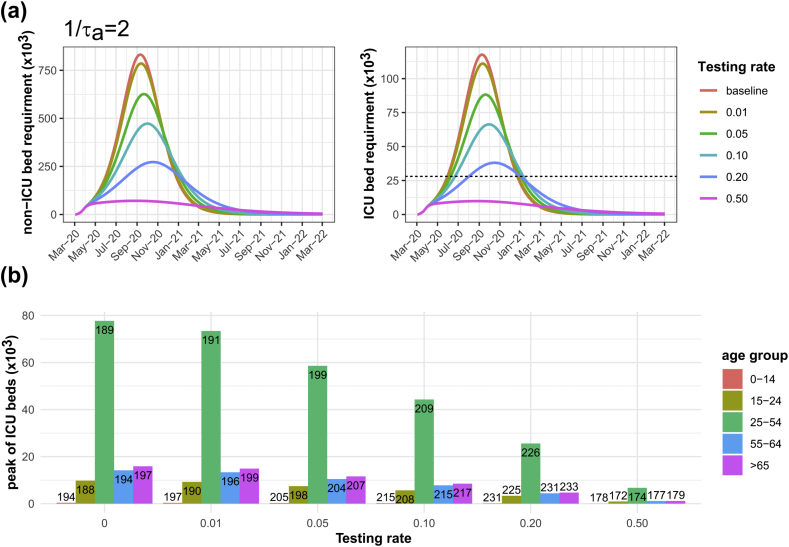


**figs2:**
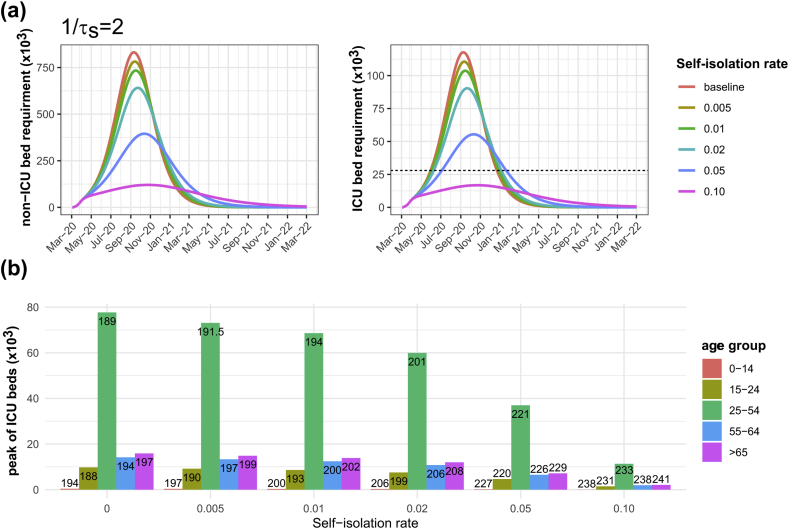


## References

[1] Guan W-j, Ni Z-y, Hu Y., Liang W-h, Ou C-q, He J-x (2020). Clinical characteristics of coronavirus disease 2019 in China. N. Engl. J. Med..

[2] Li Q., Guan X., Wu P., Wang X., Zhou L., Tong Y. (2020). Early transmission dynamics in Wuhan, China, of novel coronavirus–infected pneumonia. N. Engl. J. Med..

[3] (2020). China: First Local Transmission in the EU/EEA − Third Update [database on the Internet].

[4] Spiteri G., Fielding J., Diercke M., Campese C., Enouf V., Gaymard A. (2020). First cases of coronavirus disease 2019 (COVID-19) in the WHO European region, 24 january to 21 february 2020. Euro Surveill..

[5] Hellewell J., Abbott S., Gimma A., Bosse N.I., Jarvis C.I., Russell T.W. (2020). Feasibility of controlling COVID-19 outbreaks by isolation of cases and contacts. Lancet Glob. Health.

[6] Kucharski A.J., Russell T.W., Diamond C., Liu Y., Edmunds J., Funk S. (2020). Early dynamics of transmission and control of COVID-19: a mathematical modelling study. Lancet Infect. Dis..

[7] Zhang S., Diao M., Yu W., Pei L., Lin Z., Chen D. (2020). Estimation of the reproductive number of novel coronavirus (COVID-19) and the probable outbreak size on the Diamond Princess cruise ship: a data-driven analysis. Int. J. Infect. Dis..

[8] Russell T.W., Hellewell J., Jarvis C.I., van Zandvoort K., Abbott S., Ratnayake R. (2020). Estimating the infection and case fatality ratio for coronavirus disease (COVID-19) using age-adjusted data from the outbreak on the Diamond Princess cruise ship, February 2020. Euro Surveill..

[9] Nishiura H., Kobayashi T., Miyama T., Suzuki A., Jung S-m, Hayashi K. (2020). Estimation of the asymptomatic ratio of novel coronavirus infections (COVID-19). Int. J. Infect. Dis..

[10] Mizumoto K., Kagaya K., Zarebski A., Chowell G. (2020). Estimating the asymptomatic proportion of coronavirus disease 2019 (COVID-19) cases on board the Diamond Princess cruise ship, Yokohama, Japan, 2020. Euro Surveill..

[11] Cohen J., Kupferschmidt K. (2020). Mass testing, school closings, lockdowns: countries pick tactics in ‘war’against coronavirus. Science.

[12] Normile D. (2020). ‘Suppress and lift’: Hong Kong and Singapore say they have a coronavirus strategy that works. Science.

[13] Prem K., Liu Y., Russell T.W., Kucharski A.J., Eggo R.M., Davies N. (2020). The effect of control strategies to reduce social mixing on outcomes of the COVID-19 epidemic in Wuhan, China: a modelling study. Lancet Public Health.

[14] Bayham J., Fenichel E.P. (2020). Impact of school closures for COVID-19 on the US health-care workforce and net mortality: a modelling study. Lancet Public Health.

[15] Yang Z., Zeng Z., Wang K., Wong S.-S., Liang W., Zanin M. (2020). Modified SEIR and AI prediction of the epidemics trend of COVID-19 in China under public health interventions. J. Thorac. Dis..

[16] Hou C., Chen J., Zhou Y., Hua L., Yuan J., He S. (2020). The effectiveness of quarantine of Wuhan city against the Corona Virus Disease 2019 (COVID-19): a well-mixed SEIR model analysis. J. Med. Virol..

[17] Wu J.T., Leung K., Leung G.M. (2020). Nowcasting and forecasting the potential domestic and international spread of the 2019-nCoV outbreak originating in Wuhan, China: a modelling study. Lancet.

[18] Wang K., Ding L., Yan Y., Dai C., Qu M., Jiayi D. (2020). Modelling the initial epidemic trends of COVID-19 in Italy, Spain, Germany, and France. PloS One.

[19] Barbarossa M.V., Fuhrmann J., Meinke J.H., Krieg S., Varma H.V., Castelletti N. (2020). Modeling the spread of COVID-19 in Germany: early assessment and possible scenarios. PloS One.

[20] Khailaie S., Mitra T., Bandyopadhyay A., Schips M., Mascheroni P., Vanella P. (2021). Development of the reproduction number from coronavirus SARS-CoV-2 case data in Germany and implications for political measures. BMC Med..

[21] Population pyramids of the World from 1950 to 2100. https://www.populationpyramid.net/germany/2019/.

[22] Bhatraju P.K., Ghassemieh B.J., Nichols M., Kim R., Jerome K.R., Nalla A.K. (2020). Covid-19 in critically ill patients in the seattle region—case series. N. Engl. J. Med..

[23] Prem K., Cook A.R., Jit M. (2017). Projecting social contact matrices in 152 countries using contact surveys and demographic data. PLoS Comput. Biol..

[24] Verity R., Okell L.C., Dorigatti I., Winskill P., Whittaker C., Imai N. (2020). Estimates of the severity of coronavirus disease 2019: a model-based analysis. Lancet Infect. Dis..

[25] He X., Lau E.H.Y., Wu P., Deng X., Wang J., Hao X. (2020). Temporal dynamics in viral shedding and transmissibility of COVID-19. Nat. Med..

[26] Davies N.G., Klepac P., Liu Y., Prem K., Jit M., Pearson C.A.B. (2020). Age-dependent effects in the transmission and control of COVID-19 epidemics. Nat. Med..

[27] Bi Q., Wu Y., Mei S., Ye C., Zou X., Zhang Z. (2020). Epidemiology and transmission of COVID-19 in 391 cases and 1286 of their close contacts in Shenzhen, China: a retrospective cohort study. Lancet Infect. Dis..

[28] Ferguson N., Laydon D., Nedjati Gilani G., Imai N., Ainslie K., Baguelin M. (2020).

[29] Grasselli G., Zangrillo A., Zanella A., Antonelli M., Cabrini L., Castelli A. (2020). Baseline characteristics and outcomes of 1591 patients infected with SARS-CoV-2 admitted to ICUs of the lombardy region, Italy. JAMA..

[30] Wang D., Hu B., Hu C., Zhu F., Liu X., Zhang J. (2020). Clinical characteristics of 138 hospitalized patients with 2019 novel coronavirus–infected pneumonia in wuhan, China. JAMA.

[31] Zhou R., Li F., Chen F., Liu H., Zheng J., Lei C. (2020). Viral dynamics in asymptomatic patients with COVID-19. Int. J. Infect. Dis..

[32] Yang X., Yu Y., Xu J., Shu H., Xia Ja, Liu H. (2020). Clinical course and outcomes of critically ill patients with SARS-CoV-2 pneumonia in Wuhan, China: a single-centered, retrospective, observational study. Lancet. Respir. Med..

[33] Dudel C., Riffe T., Acosta E., van Raalte A.A., Myrskyla M. (2020). Monitoring trends and differences in COVID-19 case fatality rates using decomposition methods: contributions of age structure and age-specific fatality. PloS One.

[34] Dong E., Du H., Gardner L. (2020). An interactive web-based dashboard to track COVID-19 in real time. Lancet Infect. Dis..

[35] Cori A. (2019). R package version 2.2.

[36] Cereda D., Tirani M., Rovida F., Demicheli V., Ajelli M., Poletti P. (2020).

[37] Kucharski A.J., Klepac P., Conlan A.J.K., Kissler S.M., Tang M.L., Fry H. (2020). Effectiveness of isolation, testing, contact tracing, and physical distancing on reducing transmission of SARS-CoV-2 in different settings: a mathematical modelling study. Lancet Infect. Dis..

[38] Karin O., Bar-On Y.M., Milo T., Katzir I., Mayo A., Korem Y. (2020). Cyclic exit strategies to suppress COVID-19 and allow economic activity. medRxiv.

[39] Employment rate by age group. https://data.oecd.org/emp/employment-rate-by-age-group.htm.

[40] Brandt M. (2020). Around 34 intensive care beds per 100,000 inhabitants. https://de.statista.com/infografik/21122/anzahl-der-betten-zur-intensivmedizinischen-versorgung-in-deutschland/.

[41] Poston J.T., Patel B.K., Davis A.M. (2020). Management of critically ill adults with COVID-19. JAMA.

[42] European Health Information Gateway (2020). https://gateway.euro.who.int/en/indicators/hfa_542-6210-bed-occupancy-rate-acute-care-hospitals-only/.

[43] Rothe C., Schunk M., Sothmann P., Bretzel G., Froeschl G., Wallrauch C. (2020). Transmission of 2019-nCoV infection from an asymptomatic contact in Germany. N. Engl. J. Med..

[44] Song J.-Y., Yun J.-G., Noh J.-Y., Cheong H.-J., Kim W.-J. (2020). Covid-19 in South Korea — challenges of subclinical manifestations. N. Engl. J. Med..

[45] Chang D., Xu H., Rebaza A., Sharma L., Dela Cruz C.S. (2020). Protecting health-care workers from subclinical coronavirus infection. Lancet. Respir. Med..

[46] Giordano G., Blanchini F., Bruno R., Colaneri P., Di Filippo A., Di Matteo A. (2020). Modelling the COVID-19 epidemic and implementation of population-wide interventions in Italy. Nat. Med..

[47] (2020). Humanity tested. Nat. Biomed. Eng..

[48] Peto J. (2020). Covid-19 mass testing facilities could end the epidemic rapidly. BMJ.

[49] Kucharski A.J., Klepac P., Conlan A., Kissler S.M., Tang M., Fry H. (2020). Effectiveness of isolation, testing, contact tracing and physical distancing on reducing transmission of SARS-CoV-2 in different settings. Lancet Infect. Dis..

[50] Lu N., Cheng K.-W., Qamar N., Huang K.-C., Johnson J.A. (2020). Weathering COVID-19 storm: successful control measures of five Asian countries. Am. J. Infect. Contr..

[51] Eikenberry S.E., Mancuso M., Iboi E., Phan T., Eikenberry K., Kuang Y. (2020). To mask or not to mask: modeling the potential for face mask use by the general public to curtail the COVID-19 pandemic. Infectious Disease Modelling.

[52] Stutt R.O., Retkute R., Bradley M., Gilligan C.A., Colvin J. (2020). A modelling framework to assess the likely effectiveness of facemasks in combination with ‘lock-down’in managing the COVID-19 pandemic. Proc. Roy. Soc.A.

[53] Ngonghala C.N., Iboi E., Eikenberry S., Scotch M., MacIntyre C.R., Bonds M.H. (2020). Mathematical assessment of the impact of non-pharmaceutical interventions on curtailing the 2019 novel Coronavirus. Math. Biosci..

