# Supplementary material for: Modelling the effectiveness of intervention strategies to control COVID-19 outbreaks and estimating healthcare demand in Germany
Source: Public Health Pract (Oxf). 2021 Apr 19;2:100121. doi: 10.1016/j.puhip.2021.100121 (PMC8054549; doi:10.1016/j.puhip.2021.100121)
Supplement: Multimedia component 1 [file mmc1.docx]

**Supplementary Information**


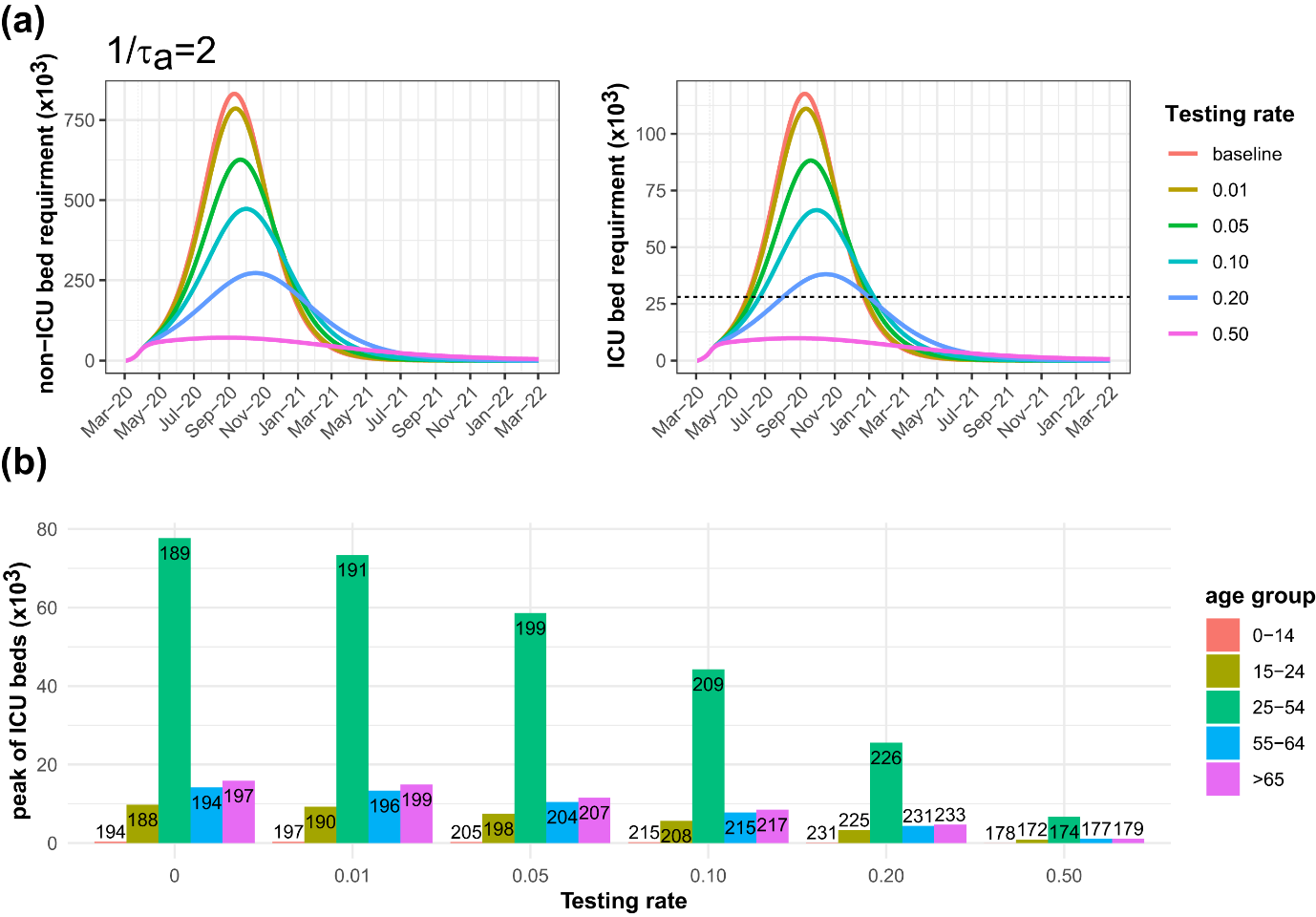


**Fig S1.** A 2-day delay in receiving testing results. Simulation outcomes for difference testing rate of incidences, non-ICU and ICU bed requirement compared to baseline (a) and age-specific ICU beds requirements at the peak with the time to peak indicated in the bars (b). The black dashed lines represent the total capacity of 28,000 ICU beds.


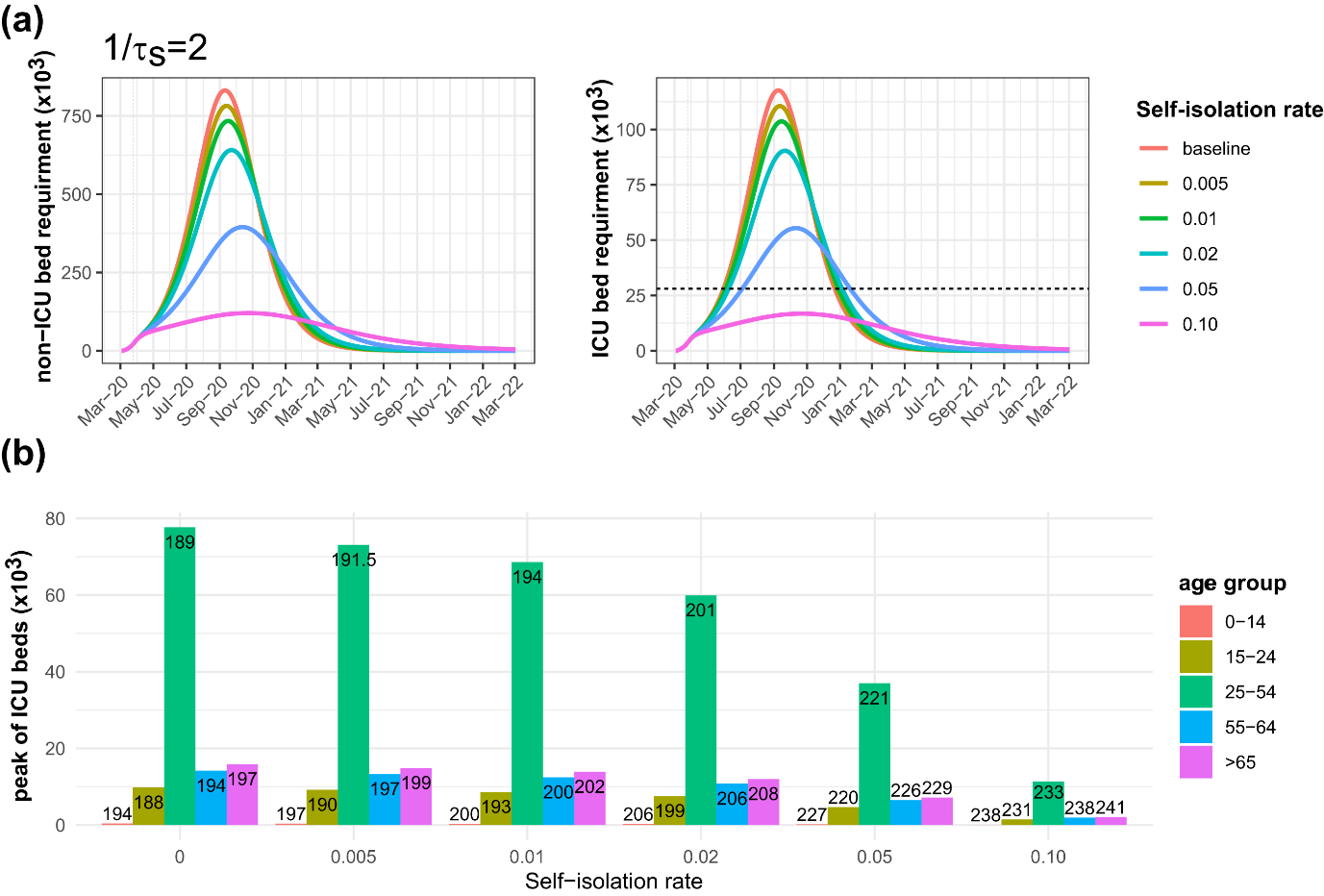


**Fig S2.** A 2-day delay until of self-isolation. Simulation outcomes for difference self-isolation rate of incidences, non-ICU and ICU bed requirement compared to baseline (a) and age-specific ICU beds requirements at the peak with time to peak indicated in the bars (b). The black dashed lines represent the total capacity of 28,000 ICU beds.

**Table S1**. Combined testing and self-isolation scenarios. The estimated number of total incidences and peak capacity requirement of ICU beds with time to peak.

| Scenarios | | | | Total Incidence | Requirement at peak | |
| --- | --- | --- | --- | --- | --- | --- |
|  |  |  |  |  | **Non-ICU** | **ICU** |
| Base | | | | 22.0 M | 832 k | 118 k |
|  | $\boldsymbol{\delta}$ | $\boldsymbol{y}$ | |  |  |  |
| $\boldsymbol{1/}\boldsymbol{\tau}_{\boldsymbol{a}}\boldsymbol{=2}$ and $\boldsymbol{1/}\boldsymbol{\tau}_{\boldsymbol{s}}\boldsymbol{=2}$ | 0.01 | - | | 21.3 M | 786 k | 111 k |
|  | 0.05 | - | | 18.8 M | 626 k | 87.5 k |
|  | 0.10 | - | | 16.1 M | 473 k | 65.8 k |
|  | - | 0.005 | | 21.2 M | 783 k | 111 k |
|  | - | 0.01 | | 20.5 M | 734 k | 104 k |
|  | - | 0.02 | | 19.1 M | 641 k | 90.4 k |
|  | 0.01 | 0.005 | | 20.6 M | 737 k | 104 k |
|  | 0.01 | 0.01 | | 19.8 M | 690 k | 97.4 k |
|  | 0.01 | 0.02 | | 18.4 M | 599 k | 84.4 k |
|  | 0.05 | 0.005 | | 18.0 M | 582 k | 81.9 k |
|  | 0.05 | 0.01 | | 17.3 M | 539 k | 75.8 k |
|  | 0.05 | 0.02 | | 15.9 M | 458 k | 64.3 k |
|  | 0.10 | 0.005 | | 15.3 M | 434 k | 60.8 k |
|  | 0.10 | 0.01 | | 14.6 M | 396 k | 55.5 k |
|  | 0.10 | 0.02 | | 13.1 M | 326 k | 45.7 k |
| $\boldsymbol{1/}\boldsymbol{\tau}_{\boldsymbol{a}}\boldsymbol{=1}$ and $\boldsymbol{1/}\boldsymbol{\tau}_{\boldsymbol{s}}\boldsymbol{=1}$ | 0.01 | | - | 20.3 M | 726 k | 102 k |
|  | 0.05 | | - | 15.1 M | 423 k | 59.3 k |
|  | 0.10 | | - | 10.5 M | 220 k | 30.7 k |
|  | - | | 0.005 | 20.4 M | 736 k | 104 k |
|  | - | | 0.01 | 18.9 M | 645 k | 90.9 k |
|  | - | | 0.02 | 16.0 M | 480 k | 67.5 k |
|  | 0.01 | | 0.005 | 18.8 M | 634 k | 89.4 k |
|  | 0.01 | | 0.01 | 17.3 M | 549 k | 77.2 k |
|  | 0.01 | | 0.02 | 14.4 M | 396 k | 55.6 k |
|  | 0.05 | | 0.005 | 13.6 M | 351 k | 49.2 k |
|  | 0.05 | | 0.01 | 12.1 M | 287 k | 40.1 k |
|  | 0.05 | | 0.02 | 9.13 M | 181 k | 25.2 k |
|  | 0.10 | | 0.005 | 8.98 M | 171 k | 23.8 k |
|  | 0.10 | | 0.01 | 7.51 M | 131 k | 18.2 k |
|  | 0.10 | | 0.02 | 4.83 M | 76.1 k | 10.5 k |

Where M is a million unit ( $\times{10}^{6}$) and k is a thousand unit ($\times{10}^{3}$)

**Table S2**. Combined testing, self-isolation, and physical distancing scenarios. The estimated number of total incidences and peak capacity requirement of ICU beds with time to peak.

| Scenarios | | | | Total Incidence | Requirement at peak | |
| --- | --- | --- | --- | --- | --- | --- |
|  |  |  |  |  | **Non-ICU** | **ICU** |
| Base | | | | 22.0 M | 832 k | 118 k |
| $\boldsymbol{1/}\boldsymbol{\tau}_{\boldsymbol{a}}\boldsymbol{=2}$ and $\boldsymbol{1/}\boldsymbol{\tau}_{\boldsymbol{s}}\boldsymbol{=2}$ | $\delta$ | $y$ | |  |  |  |
| Without physical distancing | 0.01 | 0.01 | | 18.0 M | 582 k | 81.9 k |
|  | 0.05 | 0.01 | | 17.3 M | 539 k | 75.8 k |
| Half-lockdown for 150 days  24-Mar to 20 August  (50% of work, school and other contacts) | 0.01 | 0.01 | | 18.8 M | 618 k | 87.2 k |
|  | 0.05 | 0.01 | | 14.2 M | 470 k | 66.1 k |
| Full lockdown for 150 days  24-Mar to 20 Aug  (10% of work, school and other contacts) | 0.01 | 0.01 | | 338 k | 31.9 k | 4.86 k |
|  | 0.05 | 0.01 | | 190 k | 31.8 k | 4.86 k |
| Cyclic lockdown 15 days until 17 Jan 21  (repeated lockdown and 80% of work, school and other contacts for 15 days) | 0.01 | 0.01 | | 229 k | 31.9 k | 4.86 k |
|  | 0.05 | 0.01 | | 215 k | 31.8 k | 4.86 k |
| Cyclic lockdown 30 days until 17 Jan 21  (repeated lockdown and 80% of work, school and other contacts for 30 days) | 0.01 | 0.01 | | 202 k | 31.9 k | 4.86 k |
|  | 0.05 | 0.01 | | 191 k | 31.8 k | 4.86 k |
| $\boldsymbol{1/}\boldsymbol{\tau}_{\boldsymbol{a}}\boldsymbol{=1}$ and $\boldsymbol{1/}\boldsymbol{\tau}_{\boldsymbol{s}}\boldsymbol{=1}$ | $\delta$ | | $y$ |  |  |  |
| Without physical distancing | 0.01 | | 0.01 | 13.6 M | 351 k | 49.2 k |
|  | 0.05 | | 0.01 | 12.1 M | 287 k | 40.1 k |
| Half-lockdown for 150 days  24-Mar to 20 August  (50% of work, school and other contacts) | 0.01 | | 0.01 | 14.5 M | 480 k | 67.5 k |
|  | 0.05 | | 0.01 | 1.38 M | 68.2 k | 9.69 k |
| Full lockdown for 150 days  24-Mar to 20 Aug  (10% of work, school and other contacts) | 0.01 | | 0.01 | 198 k | 31.9 k | 4.87 k |
|  | 0.05 | | 0.01 | 173 k | 31.8 k | 4.87 k |
| Cyclic lockdown 15 days until 17 Jan 21  (repeated lockdown and 80% of work, school and other contacts for 15 days) | 0.01 | | 0.01 | 216 k | 31.9 k | 4.87 k |
|  | 0.05 | | 0.01 | 201 k | 31.8 k | 4.87 k |
| Cyclic lockdown 30 days until 17 Jan 21  (repeated lockdown and 80% of work, school and other contacts for 30 days) | 0.01 | | 0.01 | 192 k | 31.9 k | 4.87 k |
|  | 0.05 | | 0.01 | 182 k | 31.8 k | 4.87 k |

Where M is a million unit ( $\times{10}^{6}$) and k is a thousand unit ($\times{10}^{3}$)
